# Identification of Key Success Factors for Private Science Parks Established from Brownfield Regeneration: A Case Study from China

**DOI:** 10.3390/ijerph16071295

**Published:** 2019-04-11

**Authors:** Xiao-Hai Weng, Yu-Ming Zhu, Xiao-Yu Song, Naveed Ahmad

**Affiliations:** School of Management, Northwestern Polytechnical University, Xi’an 710072, China; 13381606681@126.com (X.-H.W.); naveedahmad@mail.nwpu.edu.cn (N.A.)

**Keywords:** brownfield regeneration, private science parks, key success factors, Fuzzy-DEMATEL, sustainable urban development

## Abstract

Private science parks (PSPs) are infrastructure elements of national high technology industrial development zones. Increasing private capital is being invested in this field to transform abandoned factories into science parks through brownfield regeneration, which not only effectively utilizes urban space, but also greatly strengthens the power of scientific and technological innovation. The evolution of these PSPs, however, is not satisfactory, and some operation and innovation-related problems often lead to their failures. Therefore, identifying key success factors is crucial for the sustainable growth of PSPs. This study employs Fuzzy Analytic hierarchy process (FAHP) and Fuzzy-DEMATEL (Decision Making Trial and Evaluation Laboratory) methods to construct an identification model for key success factors of PSPs established from brownfield regeneration. Associated influencing factors were collected through literature analysis, on-site interviews, and questionnaire, based on which key success factors were identified. The results of the study showed that five factors—resources sharing capacity of the park, park scale, financing and financial services, legal policy services and administrative capability, and construction level of facilities in the park—are the key success factors for such PSPs. The results also provide a theoretical basis for the development of PSPs established from brownfield regeneration, and support the formulation of PSP-related policies.

## 1. Introduction

The concept of brownfield was first introduced in the “Comprehensive Environmental Response, Compensation and Liability Act” by the US Congress in 1980. The Act describes that brownfields are lands whose expansion, redevelopment, and reuse are restricted by known or suspected soil pollutants or contaminants due to hazardous waste [1]. In 2000, Alker, S. proposed that a brownfield site is any land or premises which has previously been used or developed and is not currently fully in use, although it may be partially occupied or utilized [2]. Then US EPA defined a brownfield site as real property, the expansion, redevelopment, or reuse of which may be complicated by the presence or potential presence of a hazardous substance, pollutant, or contaminant [3]. In the context of green development, a large number of enterprises were shut down, producing plentiful brownfield sites and disturbing the environmental integrity across the globe. According to the “National Soil Pollution Status Survey Bulletin” published in 2014, among the 2523 soil sampling points in the 146 surveyed industrial parks, the points with over-discharged pollutants accounted for 30% of the total [4]. With the accelerated urbanization and industrial restructuring, the planning and reuse of urban brownfields has become stringent, and the transformation from post-industrial sites to private science parks is serving as an effective approach for urban brownfield regeneration (BR). There can be different possible options to brownfield regeneration; e.g., housing development, commercial development, environmental friendly parks (sustainable parks, science parks, eco-industrial parks etc.) [5]. However, due to the increasing importance of science parks (SPs) especially in the surroundings of industrial areas, it could be an effective approach to transform brownfields (mostly existed in the industrial area) into SPs. SPs can be defined as, “A property-based development that provides housing facility and promotes the growth of enterprises and that is associated with a university (or any public or private research institute) based on proximity, ownership and governance” [6]. In 1991, the Chinese government established 27 national SPs which were regarded as very important components of the innovation system [7]. SPs had been growing rapidly in the decade following their establishment in China because of their significant role in the promotion of innovations [8]. In recent years, various types of SPs have been launched to improve the innovation capabilities of the innovation system in China. The private science park is a new member for the science park family, which is an important infrastructure element to the existing national, provincial, and municipal high-technology industries development zones in China. The rise of private science parks marks a new stage for the development of science parks in China, which is of great significance to the growth of China’s private economy and private high-technology industry. However, due to the characteristics of brownfield regeneration and the growth environment for science parks, there are still some problems in the development of these parks, such as single business model, few incubated enterprises, lack of management mechanism, and poor service quality [9]. These problems have seriously hindered the development of private science parks and have a major impact on their success. Therefore, identifying the key success factors in a comprehensive and scientific way for private science parks is critical for their development.

With the emergence of sustainable urban infrastructure development, brownfield regeneration has greatly contributed to the restoration and construction of urban environment, and how to deal with the relationship between human and nature has captured attention in earlier studies. Burke H. studied the brownfield regeneration in coalfield areas, and believed the subsurface geological information could be useful to characterize the problematic soil conditions in coalfield areas. Processing those data via 3D spatial modeling in the county of Yorkshire in England, he concluded that if decision-makers effectively use those data to identify problematic soils in the early stage of brownfield development, the time and cost spent on brownfield regeneration can be reduced [10]. Frantal B. identified the factors for success of brownfield regeneration and explored the significant similarities and differences among driving forces and obstacles in the process of brownfield regeneration under different geographical and institutional conditions [11]. Andres L. studied the tension between the mainstream cultural governance and the creative brownfield and demonstrated the importance of culture in brownfield regeneration [12]. Green T. L. quantitatively investigated the driving forces to promote successful brownfield regeneration and tested the influencing factors that can lead towards the successful brownfield regeneration [13]. Osman R. explored the factors of postsocialistic space, which were of great significance to the success of brownfield regeneration [14]. Bardos R.P. argued that, besides economic benefits, brownfield regeneration projects could create more values for stakeholders and established a brownfield opportunity matrix to identify the opportunities for creating brownfield regeneration values [15]. Zielke P. by taking a creative industrial park in Guangzhou as an example, analyzed the problems of brownfield regeneration in China based on the existing framework of brownfield management and discussed the governance model of brownfield regeneration in China [16]. Several scholars have mentioned the significance and values of creative brownfield regeneration. It is an important form for creative regeneration of urban brownfield to establish science parks through private capitals and brownfield regeneration.

Scholars have undertaken a lot of studies on science parks and analyzed the significance, values, roles, network, and performance of science parks from three perspectives: region, the park itself, and enterprises in the park. Based on literature search and balanced scorecard, Messeghem and Karim established a performance measurement model for nonprofit science parks and incubators, which provided guidance for the performance evaluation of science parks [17]. M’Chirgui and Zouhaier studied the incubation performance of science parks, and analyzed why some science parks can better support the development of scientific and technological enterprises based on the resource dependence theory [18]. Di Fatta and David analyzed the interrelationship of enterprises in science parks, and found that an enterprise that played the core role in the cooperation network in a science park, and carried out more cooperative innovation projects [19]. Wei Keat Benny Ng divided science parks into three types: research type, cooperation type, and incubation type, by investigating and analyzing 82 science parks in Europe, and compared the similarities and differences of the three types, which provided guidance for better understanding and management of science parks [20]. Based on the technology transfer mechanism, Wulung, R B Seno et al. put forward a technology selection model for science parks and analyzed the impact of technology level and technology assimilation rate on the technology selection in parks, thus guiding the technology transfer cooperation between science parks and universities [21]. Xiao Li analyzed the impact of technical support and entrepreneurship guidance provided by urban science parks at different levels on the graduation results of park enterprises, and concluded that the technical support and entrepreneurship guidance provided by urban science parks in first-tier cities had a significant positive impact on the graduation results of park enterprises, while the impact was weakened in second and third-tier cities. They also verified that the level of social and economic development and the park location had an impact on the graduation results of park enterprises [22]. Klingbeil and Caren studied the relationship between the park scale and the development of enterprises in a park. They believed that the scale had no general positive or negative effect on the development of enterprises, while the development depended on the venture team of enterprises and whether they belonged to high-tech industries [23]. Vander Straeten studied the influence of industry agglomeration in science parks on the growth and survival of enterprises, and found that the service customization strategy of science parks had a significant regulation effect on the growth and survival of enterprises [24]. Van Weele studied the insufficient resources utilization in science parks, and revealed that the park managers were unaware of the lack of resources and could not develop intangible resources by utilizing tangible resources under the short-term goal tendency. The science parks needed the assertion strategy to improve their resource utilization [25].

However, all these studies focused on the factors and performance of science parks, but did not identify the key factors for the success of parks, nor did they conduct targeted research on science parks established from private capital by using a sound and scientific methodology. For this reason, this paper studies the key success factors for private science parks established from brownfield regeneration and guides the development of those parks.

The paper is organized as follows: in the next section, we present the materials and methods used in identifying the key success factors. Subsequently, the recognition and analysis of the key success factors by means of FAHP and Fuzzy-DEMATEL methods are explained in Section 3. Finally, the results are discussed in Section 4 and the main achievements and conclusions as well as remarks are summarized in Section 5.

## 2. Materials and Methods

**Figure 1:**
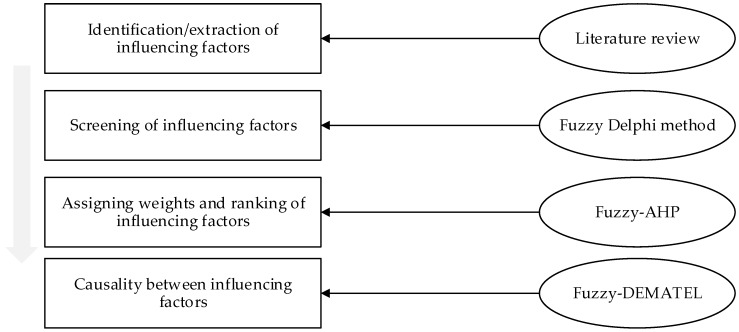
Study scheme of the paper (AHP: Analytic hierarchy process; DEMATEL: Decision Making Trial and Evaluation Laboratory).

This study integrated multiple methods to obtain the key success factors of PSPs. The research began with identifying an initial set of PSP influencing factors through a detailed and comprehensive literature review. Next, influencing factors were screened out through the Fuzzy Delphi method. Then, the influencing factors were ranked and each assigned with a weight by using Fuzzy-AHP. Finally, causality between influencing factors was calculated through Fuzzy-DEMATEL. The step-by-step scheme of the study is presented in Figure 1.

### 2.1. Extraction of Initial set of Influencing Factors through Literature Review

At the beginning, this study identified a set of influencing factors for private science parks established from brownfield regeneration by means of literature research and interviews, of which the steps are given below:

Step 1: visit the Web of Science platform and Google Scholar, and explore literature with the keywords “brownfield regeneration” and “science park/private science park/incubator”. Since the research on science parks can be dated back to the work of Monck et al. in 1998 [26], the period of the literature search was set from 1998 to 2018.

Step 2: exclude factors unrelated to the science parks by reading the abstracts of searched literature. Through an overall reading of the remainder of papers, influencing factors related to science parks were selected and form an initial set of influencing factors M.

Step 3: optimize the set M, and form another set of influencing factors N based on M, as well as based on the on-site interviews with public administration agencies, management, and enterprise owners in science parks.

### 2.2. Screening of Related Influencing Factors through Fuzzy Delphi Method

#### 2.2.1. Development of Questionnaire

Based on the set of influencing factors N, the importance of each factor, which was obtained through questionnaire survey, was evaluated, therefore forming a set of influencing factors P. The questionnaire was designed in the form of a 5-point Likert Scale, consisting of three parts—the background and purpose of the questionnaire, personal information, and a scoring table for the importance of related influencing factors. The set of factors N (31 factors) were divided into five categories: (a) construction of infrastructure in the park and its surroundings, (b) industrial chain and enterprise development, (c) capacity to support scientific & technological innovations, (d) park management and public services, and (e) government support.

People involved in the questionnaire survey included managers of science parks, enterprises in parks, faculty members from universities, and staff members of related governmental agencies. In this paper, the questionnaire was filled in through online and on-site surveys. The online survey obtained statistical data directly from the network, while the on-site survey manually collected all the questionnaires from the target people and data was entered into a statistical table, forming the complete survey data. Respondents were asked to score the importance of each influencing factor on the basis of 5-point Likert scale, where the number “1” means “very unimportant”; “2” means “unimportant”; “3” means “moderate”; “4” means “important”, and “5” means “very important”. Each item in the questionnaire relied on the opinions from experts and scholars, which was determined through repeated discussions and revisions, as well as the Delphi Method.

#### 2.2.2. Data Analysis

This study identified essential factors through Fuzzy Delphi Method and screened out a set of factors through expert opinions [27,28]. Each factor was compared with a threshold $\tilde{b}$ (where $\tilde{b}$ was the average weight $\tilde{b_{j}}$ of the initial set of all factors) to identify the essential factors. The rule to scrutinize is as follows:

(1) $$\tilde{b_{j}}\geq \tilde{b}\;=Selected,\tilde{b_{j}}<\tilde{b}\;=Rejected$$

The average score of the importance of each factor was calculated, by which the set of influencing factors P was determined.

### 2.3. Identification of Key Success Factors based on Fuzzy-AHP and Fuzzy-DEMATEL

This study made a novel contribution by integrating two methodologies: fuzzy-AHP (FAHP) and fuzzy-DEMATEL. Except for DEMATEL and AHP, many other Multiple Criteria Decision Making (MCDM) methodologies have been introduced and extensively used e.g., Analytic Network Process (ANP), Interpretive Structural Modeling (ISM), Structural Equation Modeling (SEM) and Graph theory [29,30,31,32,33]. However, the integration of FAHP and Fuzzy-DEMATEL is more advantageous for various reasons.

AHP has multiple advantages, as indicated by Saaty [34]:

- It utilizes experts’ opinion about a set of factors, and integrates statistical data.
- It merges multiple assessments and resolves the differences among them.
- It manages different kinds of relations in a network with the help of quantitative and qualitative data.
- It provides help in forecasting and strategic planning through evaluation of all crucial factors in a system.

DEMATEL is a multiple-Criteria Decision-Making (MCDM) methodology, firstly introduced by Geneva Research Centre of the Battelle Memorial institute [35]. It supports to identify interdependence among factors and visualizes the complicated and ambiguous causal associations with the help of diagraph. DEMATEL not only transforms the inter-relationships into cause and effect group with the help of matrixes, but also identifies the most important factors in a system through impact-relation diagram.

Due to the multiple benefits associated with these both methodologies, and the ease of quantifying the experts’ opinion in a way that can be measured and assessed, a combination of these methodologies should be used. A single methodology is not enough to solve such complex problems. AHP only provides the hierarchal structure of the factors, but ignores the cause and effect relationships between different factors. However, DEMATEL provides little passive approach as it only emphases on the inter-relationships and it does not provide a clear picture about importance of different coefficients. Without using the integrated methodology, the analysis of key success factors will be useless, and a wrong decision could be adopted. Therefore, an integrated methodology has been proposed to analyze the key success factors for private science parks established from brownfield regeneration. Further, the integrated methodology also combines the fuzzy theory with the traditional AHP and DEMATEL. The integrated methodology combining fuzzy theory with traditional AHP and DEMATEL is more advantageous where the absolute value 0 or 1 is replaced by a corresponding membership function in the subjective judgment data, which is more scientific as it reflects the fuzziness of human being’s subjective judgment.

Therefore, combining the weights of factors determined by the FAHP method with the influence levels of factors in the system obtained by the Fuzzy-DEAMTEL method incorporates more data and provides more reliable conclusions.

The step-by-step application of methodological approach has been described in the further sections.

#### 2.3.1. Questionnaire Design and Data Collection

##### (1) Questionnaire Design

Based on the set of influencing factors P, the relative importance between every pair of indices and the influencing levels of indexes were obtained by questionnaire. The questionnaire was divided into FAHP and Fuzzy-DEMATEL parts. The former one used Likert Scale (scaling 1–9) to judge the relative importance between factors as recommended by Saaty [34]. The Fuzzy-DEMATEL part used the Table 1 to collect the relationship between factors and obtained the expert judgment matrix, and then obtained the triangular fuzzy number between every two factors.

##### (2) Questionnaire Distribution and Collection

Five experts engaged in the field of science parks were invited to participate in the questionnaire survey, including two professors and scholars from universities, two park managers, and one from a government agency (Table 2).

#### 2.3.2. Assigning Weights to Influencing Factors

This study used the FAHP method to determine the weight of each index in the set of influencing factors P. Despite the multiple advantages of AHP as mentioned in Section 2.3, some researchers, however, pointed out that there are still some deficiencies in this method. For example, only memberships 0 and 1 are used to construct the judgment matrix for the comparison between every pair of indices, where the values are so absolute and unscientific and cannot reflect the fuzziness of a human being’s subjective judgment; the consistency test of the judgment matrix lacks scientific basis but with a complicated process; and the criterion to check if the judgment matrix is consistent is defined as CR < 0.1, which lacks scientific support.

In order to solve these shortcomings, Van Loargoven, a Dutch scholar, proposed a method in 1983 which utilizes triangular fuzzy numbers to make fuzzy comparison and judgment between indexes [36]. Based on the calculations of triangular fuzzy numbers and the principle of least square method, the ranking and weights of factors are obtained. Finally, the traditional AHP method is extended to the fuzzy analytic hierarchy process (FAHP) which can be used in the fuzzy environment, and overcomes those weaknesses well.

The weights are calculated as follows:

##### (1) Define the Triangular Fuzzy Number

The triangular fuzzy number is defined as follows: assuming the fuzzy number on the domain of discourse R is $\;\hat{A}\;$, if by making R→[0,1], the membership function $\mu_{\hat{A}}\left(x\right)$ of $\hat{A}$ is expressed as:

(2) $$\mu_{\hat{A}}\left(x\right)=\{\begin{array}{cc} \frac{x}{m-l}-\frac{l}{m-1}, & l\leq x\leq m\\ \frac{x}{m-u}-\frac{u}{m-u}, & m\leq x\leq u\\ 0, & x<l\;or\;\;x>u\; \end{array}$$

**Figure 2:**
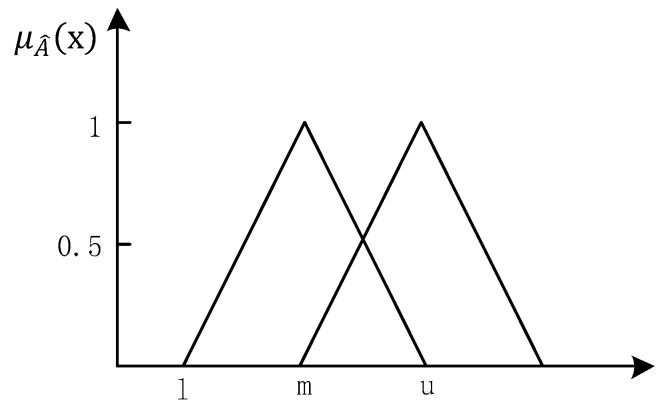
Definition of triangular fuzzy number.

Then $\hat{A}$ is called as a triangular fuzzy number, which is generally denoted as (l, m, u). As shown in Figure 2, l and u represent the upper and lower bounds of the fuzzy number, respectively, and $\mu_{\hat{A}}\left(x\right)$ is the triangular fuzzy function.

##### (2) Establish the Relationship between Semantic Scale and Triangular Fuzzy Number.

During the development of the questionnaire, the semantic scale of expert evaluation can be divided according to the relative importance between compared factors. Generally, the scaling 1–9, which is commonly used in AHP, can be employed for reference. Its usual conversion relationship is shown in Table 3.

##### (3) Development of Judgment Matrix and Determination of Initial Weights.

The triangular fuzzy number is $\hat{A}={\left(\;a_{ij}\right)}_{n\times m\;}$, $a_{ij}=\left(l_{ij},m_{ij},u_{ij}\right)$. When there are T experts making the judgment together, $a_{ij}$ is the comprehensive triangular fuzzy number, and $a_{ij}$ is expressed as:

(3) $$a_{ij}=\frac{1}{T}\left(a_{ij}{}^{1}⊕a_{ij}{}^{2}⊕a_{ij}{}^{3}⊕\ldots a_{ij}{}^{T}\right)$$

The fuzzy value (initial weight) of the i^th^ element in the k^th^ layer is defined as $\;F_{i}^{k}$:

(4) $$F_{i}^{k}=\sum_{j=1}^{n}a_{ij}^{k}/\left(\sum_{i=1}^{m}\sum_{j=1}^{n}a_{ij}^{k}\right)$$

##### (4) Defuzzify to Obtain the Standard Weight.

When ${\hat{A}}_{1}=\left(l_{1},m_{1},u_{1}\right)$ and ${\hat{A}}_{2}=\left(l_{2},m_{2},u_{2}\right)$, the possibility degree of ${\hat{A}}_{1}\geq {\hat{A}}_{2}$ is defined by the triangular fuzzy function as:

(5) $$\vartheta \left({\hat{A}}_{1}\geq {\hat{A}}_{2}\right)=\sup_{x\geq y}\left[\min(u_{{\hat{A}}_{1}}\left(x\right),\left(u_{{\hat{A}}_{2}}\left(y\right)\right)\right]$$

(6) $$\vartheta \left({\hat{A}}_{1}\geq {\hat{A}}_{2}\right)=\{\begin{array}{cc} 1 & m_{1}\geq m_{2}\\ \frac{l_{2}-u_{1}}{\left(m_{1}-u_{1}\right)-\left(m_{2}-l_{2}\right)} & m_{1}\leq m_{2},\;u_{1}\geq \;l_{2}\;\\ 0 & other\;conditions \end{array}$$

Where, $\vartheta_{ij}\left({\hat{A}}_{ij}\geq {\hat{A}}_{i1},{\hat{A}}_{i2}\ldots {\hat{A}}_{in}\right)=\min\vartheta_{ij}\left({\hat{A}}_{ij}\geq {\hat{A}}_{ij}\right),j=1,2,\ldots n$

Definition: the possibility degree that a fuzzy number is larger than the other k fuzzy numbers is taken as the standard weight of this factor.

Then, the standard weight corresponding to the influencing factor at the lowest level is obtained.

(7) $$\omega_{ij}=\frac{\vartheta_{ij}}{\sum_{j=1}^{n}\vartheta_{ij}}$$

##### (5) Total Weights of Hierarchy to Determine the Final Weight.

By multiplying $\omega_{ij}$—the standard weight corresponding to the influencing factor at the lowest level, by $W_{i}$—the weight of the corresponding factor at the upper level, the final weight of the factor in the hierarchical structure $W_{ij}$ is obtained.

(8) $$W_{ij}=\omega_{ij}\times W_{i}$$

#### 2.3.3. Comprehensive Levels of Influencing Factors

The DEMATEL method was used to analyze and calculate the factors by graph theory and matrix tools, thus obtaining the logical relationship among all the indices. By calculating the influencing degree and the degree of being influenced for each index, the causal relationship among the indexes and the position in the system were determined. Therefore, the DEMATEL method is an effective method for analysis and identification of factors and setting of index weights, which makes full use of the experience and knowledge of experts. Considering that the judgment of experts on the interrelationship among indices based on their own experience and knowledge was characterized by subjectivity and fuzziness, fuzzy mathematics was introduced to fuzzify their judgment. In this study, triangular fuzzy numbers were used to convert the experts’ judgment values to fuzzy numbers. According to the congruent relationship between the linguistic variables given by Li and the membership function of the triangular fuzzy number, the five judgment levels were transformed into triangular fuzzy numbers, and their matrix was used as the initial matrix for DEMATEL calculation to compute the weights of indexes [37]. This is the Fuzzy-DEMATEL method. It is a more complicated method that evolved from the DEMATEL method and defuzzification method. See the following steps for its implementation:

(1) According to the conversion relationship between semantic evaluation and triangular fuzzy number in Table 1, the fuzzy influence matrix of related influencing factors was investigated by questionnaire: $\tilde{A}={\left[{\tilde{a}}_{ij}\right]}_{n\times n}$. Define ${\tilde{a}}_{ij}=\left(l_{ij},m_{ij},u_{ij}\right)$ to indicate the expert judgment data representing the relationship between factors  i and j.
(2) Use the CFCS method for defuzzification to obtain the direct influence matrix.
(3) Standardize the direct influence matrix directly to get the standard direct influence matrix X, so  X  is:
  (9) $$X={\left[x_{ij}\right]}_{n\times n},0\leq x_{ij}\leq 1$$
(4) Compute the comprehensive influence matrix T
  (10) $$T=X{\left(I-X\right)}^{-1},T={\left[t_{ij}\right]}_{n\times n}(i,j=1,2,3\ldots \ldots n)$$
(5) Calculate the centrality and causality for each element.
  (11) $$D={\left[\sum_{i=1}^{n}t_{ij}\right]}_{n\times 1}={\left[t_{i}\right]}_{n\times 1},\;R={\left[\sum_{j=1}^{n}t_{ij}\right]}_{1\times n}={\left[t_{j}\right]}_{1\times n}$$

The centrality $M_{i}=D_{i}+R_{i}$ indicates the influencing degree of a factor on the whole system; and the causality $N_{i}=D_{i}-R_{i}$ means the influence trend of a factor on the whole system. $N_{i}>0$ states this factor is a cause factor leading to active influence on other factors, whereas $N_{i}<0$ means this factor is a resulting factor affected by other factors.

## 3. Results

### 3.1. Extraction of Alternative Influencing Factors

This study identified the alternative influencing factors by literature review. The identified influencing factors were further analyzed and summarized based on their similarities and relevance. By communicating with experts in related fields, this study established a statistical table for the alternative influencing factors, as shown in Table 3, including five level-2 factors and 31 level-3 factors. The five level-2 factors are: construction of infrastructure in the park and its surroundings, industrial chain and enterprise development, capacity to support scientific and technological innovations, park management and public services, and government support.

### 3.2. Identification of Related Influencing Factors

After a two-month survey, 143 questionnaires were collected. By eliminating those questionnaires where respondents clearly indicated that they did not know about brownfield regeneration or science parks, 131 valid questionnaires were obtained, of which the validity rate was 91.6%. Of those 131 valid questionnaires, 46.6% had “general understanding” on science parks and 53.4% had “very good understanding” (Table 4). Among the valid questionnaires, 11 were collected from government agencies, accounting for 8.4%; 74 from science parks, accounting for 56.5%; and 46 from universities, accounting for 35.1% (Table 5).

#### 3.2.1. Data Processing

The reliability and validity of the questionnaire were tested by the statistical software SPSS 23.0 (SPSS Inc., Chicago, IL, USA). As can be seen from Table 6, the overall test results of the questionnaire showed that the Cronbach’s Alpha reliability coefficient was 0.941; the KMO (Kaiser-Meyer-Olkin) value was 0.912; and the approximate chi-square value of Bartlett’s Test of Sphericity was 2492.87 (degree of freedom was 435), reaching the significance level (*p* < 0.001). The overall questionnaire data fell within appropriate criteria and meets the data processing requirements.

#### 3.2.2. Preliminarily Screen for Influencing Factors based on the Fuzzy Delphi Method

Taking the average of expert evaluation data as the basis for screening the influencing factors and following the criteria recommended by Ahmed et al. [27], two factors (Level of competition of enterprises in the park, Level of internationalization of the park) were eliminated because they did not meet the above mentioned criteria in Section 2.2. Then, a set comprising 29 influencing factors was obtained, as shown in Table 7.

### 3.3. Assigning Weights to Influencing Factors

#### 3.3.1. Establish a Structure Model of AHP

According to the set of influencing factors retained in Table 7 for private science parks, the level-1 factors, as the factors for the success of PSPs, were determined. The level-2 factors are: construction of infrastructure in the park and its surroundings, industrial chain and enterprise development, capacity to support scientific & technological innovations, park management and public services, and government support. Moreover, there is a total of 29 level-3 factors, as listed in Table 8.

#### 3.3.2. Establish an Expert Evaluation Group and Conduct a Questionnaire Survey

In this paper, expert evaluation data was collected by means of a questionnaire survey. A group composed of five experts who were familiar with private science parks was set up, including two professors from universities, two staff members from private science parks, and one administrative staff from government agency. The survey was carried out via a paper questionnaire, which introduced the background and purpose at the beginning. Once the questionnaire was clearly explained to the five experts, each of them started to fill a copy of the questionnaire and marked the relative importance between every two factors compared.

#### 3.3.3. Construct a Fuzzy Judgment Matrix

Based on personal experience, the experts filled the questionnaire with the importance code between every two factors compared. With the filled questionnaires collected, a fuzzy judgment matrix was constructed according to the conversion relationship between the expert semantic evaluation and the triangular fuzzy number in Table 1. Calculate the triangular fuzzy numbers corresponding to the five questionnaires according to the calculation steps in Section 2.3.2 to finally obtain the weight of each index, as shown in Table 8.

### 3.4. Identification of Key Success Factors

**Figure 3:**
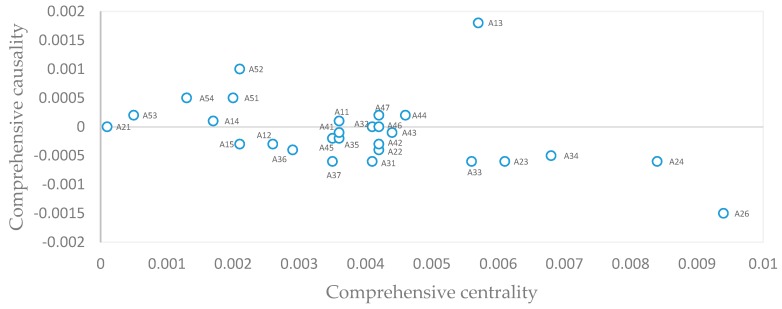
Distribution of comprehensive influencing degrees of factors.

By using EXCEL software, data processing and matrix operation were implemented through the calculation steps in Section 2.3.3. The results are shown in Table 8 and Figure 3:

## 4. Discussion

In this paper, based on the FAHP and Fuzzy-DEMATEL methods, a model to identify key success factors for the PSPs was developed, of which the principles are as follows: first of all, select and screen out the key influencing factors from the set of factors whose comprehensive causality is bigger than 0. The comprehensive causality reflects the influence trend of a factor on the whole system. Its positive value means a cause factor, which shows that the factor has an active influence on the other factors in the system. Cause factors are located at the bottom of the sequence of factors, with the deepest influence on the success of the system. Next, in the set of factors with comprehensive causality bigger than 0, select the factors with a bigger comprehensive centrality value as key influencing factor. If a factor has a higher comprehensive centrality, its influence on the whole system is more significant, to which the managers should pay attention. Finally, according to the theoretical basis corresponding to the key success factors, Pareto’s law applies. That is to say, only about 20% of all the related influencing factors in a set play an important role and can be considered as key success factors.

According to the distribution of comprehensive influencing degrees of factors in Figure 3, there are ten cause factors whose comprehensive causalities are bigger than 0. They are: construction level of facilities in the park A11, park scale A13, regional traffic convenience A14, resources sharing capability of the park A25, financing and financial services A44, legal policy services and administrative capability A47, land use policy support A51, administrative service support A52, tax funds support A53, and organizational leadership support A54. In the set of factors with comprehensive causality bigger than 0, the factors with the comprehensive centrality higher than that of the other factors are: resources sharing capability of the park A25, park scale A13, financing and financial services A44, legal policy services and administrative capability A47, and construction level of facilities in the park A11. Therefore, according to Pareto’s law, these five factors are the key factors for the success of private science parks established from brownfield regeneration.

Resource sharing capability of the park (A25) emerged as the key influencing factor, therefore, when choosing among PSPs, enterprises highly emphasize the sharing of resources in the park. The resources sharing capability of a PSP is vital for its success. On the one hand, the resources sharing can reduce the operation cost of enterprises in the park by sharing facilities such as laboratories, conference rooms and restaurants [53]; on the other hand, the cooperation among enterprises in the park can promote knowledge sharing and improve the innovation capability [48]. Further, park scale (A13) is one of the key influencing factors while considering PSPs as an option of brownfield regeneration. The park scale can promote the performance of the enterprises in the park. Enterprises in a large park have better performance than those in a small park [41]. Moreover, a large park is more likely to receive preferential policies from the government, and provide university–industry cooperation opportunities for enterprises in the park. Financial problems is a serious issue faced by the enterprises in PSPs [58]. The parks play a role of helping the enterprises to obtain investment from the government or other investment organizations [47]. Therefore, the financing and financial service capabilities of PSPs are crucial to the survival and development of enterprises in parks and are also critical to the success of PSPs. Most enterprises in science parks are high-tech enterprises, of which the most important asset is the intellectual property right. How to guarantee their intellectual property rights and other rights by laws is of great significance to the survival and development of enterprises there. Thus, legal policy services and administrative capability is one of the key factors to ensure the success of parks. The safety of the science parks established from brownfield regeneration affects an enterprise’s choice. The construction level of facilities in the park is vital to the success, as many scholars have discussed the importance of the location and environment of a park to its success. Good construction of facilities, convenient traffic, and satisfactory office environment, which affect the life quality of employees, are keys for attracting enterprises, and are indispensable factors for the success of parks [38,40,54,59].

At the same time, the development of China’s PSPs also verified the research conclusions. Shanghai’s PSPs are in a leading position in China. By the end of 2016, the number of PSPs in Shanghai had reached 76, most of which were built through brownfield regeneration, and the number keeps increasing every year. Since the brownfield regeneration significantly impacts the construction and the reputation of PSPs, most PSPs have difficulties in operations due to problems such as facilities construction, resource sharing, financing and financial services, and intellectual property rights. According to our interviews with park managers, business managers in the park, and administrative staff from government, the key factors for the success of PSPs are the resource sharing capability of the park, park scale, financing and financial services, legal policy services and administrative capability, and construction level of facilities in the park. Therefore, the research results have identified the key factors of such PSPs. PSP managers can enhance the park’s competitiveness through analyzing shortcomings of the park based on the research results.

## 5. Conclusions

Abandoned factories occupying advantageous positions in cities cannot produce economic and social value but exacerbate environmental health. Private capital can transform abandoned land into private science parks via brownfield regeneration, which greatly complements innovative elements and improves the urban ecological environment. Those parks, however, are faced with difficulty due to the deficiencies in resources and management. Consequently, this study established key factors for the success of PSPs constructed from brownfield regeneration by using literature research, and FAHP and DEMATEL methods. Through a comprehensive analysis of the existing literature about science parks, a preliminary set of influencing factors affecting the development of private science parks were identified. After interviews with experts, five level-2 factors and 31 level-3 factors were screened out. The five level-2 factors are: construction of infrastructure in the park and its surroundings, industrial chain and enterprise development, capacity to support scientific & technological innovations, park management and public services, and government support. Using a questionnaire survey, two factors (level of competition of enterprises in the park and level of internationalization of the park) were removed, thus forming a set of factors, which comprises 29 level-3 factors related to PSPs. Based on this set, the FAHP and Fuzzy-DEMATEL methods were synthetically used to construct an identification model for key success factors of PSPs established from brownfield regeneration. The FAHP method was for assigning weights to influencing factors, while the Fuzzy-DEMATEL method was used for the calculation of comprehensive causality and centrality of factors. The relative importance data of each influencing factor was collected through a questionnaire survey, thus computing the weight, comprehensive causality and comprehensive centrality of each factor. In consideration of the comprehensive causality and centrality of each factor, five key success factors were finally determined, which were resources sharing capability of the park, park scale, financing and financial services, legal policy services and administrative capability, and construction level of facilities in the park. The importance of the key success factors was explored through analysis and discussion, and the direction of future research regarding PSPs established from brownfield regeneration was put forward. The research results of this paper guide government regulators and PSP managers.

This paper studied the key factors for the success of PSPs established from brownfield generation, identified the key factors influencing the success, and guided the development of such PSPs. Science parks play an active role in promoting regional growth. There are more and more private entrepreneurs establishing science parks or incubators through urban brownfield regeneration, which also provides a new path for brownfield regeneration. Some problems, however, remain in such developments, related to business incubation, sustainable development, and innovation efficiency, which undermine the survival and evolution of a science park. As a result, the follow-up research will focus on PSPs established from brownfield regeneration once again to deepen the discussion on their development mode, analyze their innovation network, and explore methods to improve their innovation capability. In addition, while brownfield has a stigma effect [60], the good reputation of a science park has major appeal for enterprises [41,48]; therefore, how such science parks establish and maintain their reputation and social image is also a topic to be studied.

## Figures and Tables

**Table 1:** Triangular fuzzy numbers.

Influencing Level	Score Code	Triangular Fuzzy Number
No influence	0	(0, 0, 0.25)
Very weak influence	1	(0, 0.25, 0.5)
Weak influence	2	(0.25, 0.5, 0.75)
Strong influence	3	(0.5, 0.75, 1)
Very strong influence	4	(0.75, 1, 1)

**Table 2:** Information of experts.

Expert	From	Position/Title	Knowledge about BR	Knowledge about SPs
E1	Industry	PSP Manager	Know well	Know well
E2	Industry	PSP Manager	Know well	Know well
E3	Government	Administrative Manager	Know well	Know well
E4	University	Professor	Know well	Know well
E5	University	Professor	Know well	Know well

PSP: Private science park; BR: brownfield regeneration; SPs: science parks.

**Table 3:** Conversion relationship between semantic evaluation and triangular fuzzy number.

Importance	Scale	Description	Triangular Fuzzy Number
$\hat{1}$	Equally important	The two factors provide the same importance	(1, 1, 1)
$\hat{3}$	Slightly more important	One factor is slightly more important than the other.	(2, 3, 4)
$\hat{5}$	Obviously more important	One factor is obviously more important than the other.	(4, 5, 6)
$\hat{7}$	Much more important	One factor is much more important than the other.	(6, 7, 8)
$\hat{9}$	Extremely more important	One factor is extremely more important than the other.	(8, 9, 11)
$\hat{2}$,$\hat{4}$,$\hat{6}$,$\hat{8}$	Median	Median of above adjacent judgments	

**Table 4:** Number of questionnaires on understanding of science parks (SPs).

Understanding of SPs	Number of Questionnaires	Proportion
general understanding	61	46.6%
very good understanding	70	53.4%
Total	131	100%

**Table 5:** Number of questionnaires by respondent source.

Questionnaires Source	Number of Questionnaires	Proportion
government agencies	11	8.4%
science parks	74	56.5%
universities	46	35.1%
Total	131	100%

**Table 6:** Kaiser–Meyer–Olkin (KMO) and Bartlett test.

Sampling Adequacy Measure	Value
Cronbach’s Alpha	0.941
Kaiser–Meyer–Olkin (KMO)	0.912
Bartlett test of sphericity	
Approx. Chi-Square (x^2^)	2492.87
Degree of Freedom (df)	435
Significance level	0.000

**Table 7:** Related influencing factors.

Level 2 Factor	Level 3 Factor	Source	Result
Construction of infrastructure in the park and its surroundings	Construction level of facilities in the park	[38,39]	Selected
	Maturity level of commercial facilities	[38,39,40]	Selected
	Park scale	[41]	Selected
	Regional traffic convenience	[39,42]	Selected
	Regional ecological environment level	[43,44]	Selected
Industrial chain and enterprise development	Industry agglomeration ability of the park	[45]	Selected
	Industrial adaptability of the park (with regional industries)	[46]	Selected
	Level of competition of enterprises in the park	FDM	Rejected
	Financing capacity of the park	[47]	Selected
	Resources integration capability of the park	[48]	Selected
	Resources sharing capability of the park	[48]	Selected
	Enterprise incubation capability	[49]	Selected
Capability to support scientific & technological innovations	Capability to support fast iteration of enterprise products	[47]	Selected
	Richness of regional scientific research elements	[50]	Selected
	Capability to support university-industry cooperation	[48]	Selected
	Capability of acquiring senior human resources	[50]	Selected
	Technological intermediary service capability	[48]	Selected
	Construction of innovation and entrepreneurship cultures in the park	[47]	Selected
	Level of internationalization of the park	[51]	Rejected
	Capability to support the management of intellectual properties	[52]	Selected
Park management and public services	Park management capability	[39]	Selected
	Park service level	[39,53]	Selected
	Capability of talent introduction and resettlement in the park	[50]	Selected
	Financing and financial services	[47]	Selected
	Capability to support market development	[48]	Selected
	Structure of employees in the park	FDM	Selected
	Legal policy services and administrative capability	[47]	Selected
Government support	Land use policy support	[44]	Selected
	Administrative service support	[54,8]	Selected
	Tax funds support	[55,56,57]	Selected
	Organizational leadership support	[45]	Selected

**Table 8:** Comprehensive influence degree.

Level-2 Index	Level-3 Index	Weight $W_{ij}$	Influence Degree D	Degree of being Influenced R	Centrality M	Causality N	$\mathrm{Comprehensive}\;\mathrm{Centrality}\;O_{i}$	Comprehensive $\mathrm{Causality}\;P_{i}$
Construction of infrastructure in the park and its surroundings (A1)	Construction level of facilities in the park (A11)	0.0349	0.0529	0.0500	0.1029	0.0030	0.0036	0.0001
	Maturity level of commercial facilities (A12)	0.0311	0.0364	0.0476	0.0840	−0.0112	0.0026	−0.0003
	Park scale (A13)	0.0408	0.0924	0.0482	0.1406	0.0442	0.0057	0.0018
	Regional traffic convenience(A14)	0.0293	0.0311	0.0277	0.0588	0.0034	0.0017	0.0001
	Regional ecological environment level(A15)	0.0385	0.0236	0.0301	0.0537	−0.0065	0.0021	−0.0003
Industrial chain and enterprise development (A2)	Industry agglomeration ability of the park(A21)	0.0002	0.0810	0.2370	0.3180	−0.1559	0.0001	0.0000
	Industrial adaptability of the park (with regional industries) (A22)	0.0289	0.0659	0.0801	0.1461	−0.0142	0.0042	−0.0004
	Financing capacity of the park(A23)	0.0485	0.0572	0.0695	0.1267	−0.0124	0.0061	−0.0006
	Resources integration capability of the park (A24)	0.0569	0.0679	0.0789	0.1468	−0.0111	0.0084	−0.0006
	Resources sharing capability of the park (A25)	0.06	0.2043	0.0790	0.2833	0.1254	0.0170	0.0075
	Enterprise incubation capability (A26)	0.0633	0.0619	0.0860	00.1479	−0.0241	0.0094	−0.0015
Capability to support scientific & technological innovations (A3)	Capability to support fast iteration of enterprise products (A31)	0.0362	0.0493	0.0652	0.1145	−0.0159	0.0041	−0.0006
	Richness of regional scientific research elements(A32)	0.0394	0.0524	0.0526	0.1050	−0.0002	0.0041	0.0000
	Capability to support university-industry cooperation(A33)	0.0421	0.0600	0.0731	0.1331	−0.0131	0.0056	−0.0006
	Capability of acquiring senior human resources (A34)	0.0502	0.0630	0.0731	0.1361	−0.0101	0.0068	−0.0005
	Technological intermediary service capability (A35)	0.0324	0.0526	0.0582	0.1109	−0.0056	0.0036	−0.0002
	Construction of innovation and entrepreneurship cultures in the park (A36)	0.026	0.0480	0.0629	0.1109	−0.0149	0.0029	−0.0004
	Capability to support the management of intellectual properties (A37)	0.0406	0.0361	0.0511	0.0872	−0.0149	0.0035	−0.0006
Park management and public services (A4)	Park management capability(A41)	0.0235	0.0735	0.0795	0.1530	−0.0060	0.0036	−0.0001
	Park service level (A42)	0.0294	0.0666	0.0760	0.1426	−0.0095	0.0042	−0.0003
	Capability of talent introduction and resettlement in the park (A43)	0.0331	0.0657	0.0673	0.1329	−0.0016	0.0044	−0.0001
	Financing and financial services (A44)	0.0403	0.0586	0.0544	0.1130	0.0042	0.0046	0.0002
	Capability to support market development (A45)	0.0335	0.0502	0.0555	0.1056	−0.0053	0.0035	−0.0002
	Structure of employees in the park (A46)	0.0385	0.0555	0.0544	0.1098	0.0011	0.0042	0.0000
	Legal policy services and administrative capability(A47)	0.0371	0.0587	0.0533	0.1121	0.0054	0.0042	0.0002
Government support (A5)	Land use policy support (A51)	0.0261	0.0476	0.0275	0.0751	0.0201	0.0020	0.0005
	Administrative service support (A52)	0.0234	0.0655	0.0240	0.0894	0.0415	0.0021	0.0010
	Tax funds support (A53)	0.0048	0.0690	0.0257	0.0947	0.0433	0.0005	0.0002
	Organizational leadership support (A54)	0.0111	0.0800	0.0392	0.1193	0.0408	0.0013	0.0005
